# Is generative AI use causing mental health problems?

**DOI:** 10.1186/s44263-026-00320-8

**Published:** 2026-08-25

**Authors:** Christian Montag, Benjamin Becker

**Affiliations:** 1 Centre for Cognitive and Brain Sciences, Institute of Artificial Intelligence and Brain Sciences, University of Macau, Macau SAR, China; 2 Department of Computer Science, Faculty of Information Science and Computing, University of Macau, Macau SAR, China; 3 Department of Psychology, Faculty of Social Sciences, University of Macau, Macau SAR, China; 4 MIND & AI Lab, Department of Psychology, The University of Hong Kong, Hong Kong SAR, China; 5 SRT AI, Society & Social Dynamics, Faculty of Social Sciences, The University of Hong Kong, Hong Kong SAR, China

Generative artificial intelligence (GenAI) has prompted concerns about detrimental mental-health effects including AI Psychosis, cognitive decline and addictive behaviors. A complex interaction of genetic and environmental factors pave the way for the rise of psychopathologies. Therefore, understanding the health effects of GenAI needs a multifactorial instead of mono-causal perspective.

Since the launch of ChatGPT in November 2022 millions of users started to interact with generative artificial intelligence (GenAI). While both the initial GenAI use and related scientific conversations have been focused on functional job support, GenAI use patterns and discussions have increasingly shifted toward social–emotional support and health-related inquiries.

Interactions with AI on a personal and social-emotional level led to several high-profile reports on suicides after intimate conversations with AI companions or media reports on individuals spiraling into psychotic delusions (“AI Psychosis”). This is potentially related to the tendency of the models to default to sycophancy, that is to agree with, flatter or validate users even when their reasoning is wrong (for a critique of the “AI Psychosis” concept see here [1]). These sycophantic characteristics of AI systems might promote human interactions with the systems, in particular for vulnerable groups.

As with most technologies hitting the mass markets, and with the rise of GenAI, the question arises whether this technology may amplify pre-existing mental health issues, promote dysfunctional use in vulnerable individuals, and may even cause mental health disorders. In general, it is helpful to remind researchers and the wider public that moral panics about the detrimental impact of emerging technology on humans and society represent a “typical” response to new technological developments [2]. This was not different with the radio, the comic book, television, computer games or social media. But sometimes the arising concerns might be valid: for instance, Gaming Disorder (video game addiction) has recently been officially recognized by the World Health Organization (WHO) as a mental health condition (https://www.who.int/standards/classifications/frequently-asked-questions/gaming-disorder). While the debate of the nature of excessive social media use is still ongoing [3], emerging discussions across scientific communities are now focusing on phenomena such as “ChatGPT addiction”, with some researchers arguing against the addiction-framing of excessive GenAI use [4].

Indeed, recent research indicates that characterizing excessive GenAI use and potential detrimental consequences represents a complex endeavor: For instance the same AI-model can lead to *instrumental* and/or *relationship dependencies* [5]. Hence, different forms of GenAI use motivations and use patterns relate to different mental health and brain structural signatures [6]: Instrumental dependency could mean that users (excessively) rely on the power of GenAI supporting job related tasks for decision making, and while this comes with great opportunities, the problem of cognitive outsourcing has also been highlighted [7]: Some researchers fear that not practicing one’s own cognitive skills by delegating all tasks to an AI will be detrimental for cognitive functioning. Regarding relationship dependency, it could refer to heavily relying on GenAI as a substitute for human relationships. This might be the case for some people, particularly when considering the sycophantic ways of some AI’s responding to humans. But it should not be forgotten that large language model-(LLM)-based GenAI-interactions lack social touch and therefore are highly imperfect as a substitute for human-human interactions. From our view, the need for social interactions cannot be fulfilled by a GenAI companion in a satisfactory way in the long run. Recent research also shows that even texting with a random person is more effective in reducing loneliness than interacting with an AI chatbot called Sam [8].

Coming back to the addiction question: Can excessive reliance on GenAI be described as an addictive behavior—or may the excessive GenAI use promote the development and maintenance of other mental health conditions? Currently this remains an open question. One line of research should seek to understand if WHO’s Gaming Disorder criteria can be transferred to excessive use of GenAI. Following these criteria clinicians would need to see that persons prioritize use over other relevant daily activities, loss of control over GenAI use and keep on using GenAI despite negative experiences in everyday life. Most importantly, one also would need to register functional impairments. In other words, one would like to set the bar high and suggest that the excessive use of GenAI would need to result in dramatic consequences for the user to not over-pathologize everyday life behavior. One could imagine that this would mirror the detrimental emotional impact of a break-up of a romantic relationship due to “falling in love” with an AI. Or one could imagine that someone would not trust their own cognitive judgements due to constant GenAI use and is becoming unable to do a job without the support of the AI—perhaps even resulting in job loss.

This being said, just saying that interactions with GenAI are unhealthy or lead to psychopathology is likely an exaggeration. From many years of research, we know that both nature and nurture play a role in the rise of psychopathology. One factor alone such as use of GenAI will not be sufficient to explain why a person develops a mental health problem. Genetics, personality, stress, social support, etc. all play a role and should be factored in. These factors dynamically interact with each other and technology use. This makes it so hard to clearly point to the culprit triggering a mental disorder [1]. Further, it is of importance to consider GenAI use motives. A recent study reported that “a compensatory–affective system positively associated with problematic ChatGPT use (PCU), social anxiety, and digital addiction” while “a competence–adaptive system associated with higher self-esteem and social support” [9] (see abstract). Hence, use motives—no matter if distinguished in instrumental vs. relationship dominant use or being named competence-adaptive vs. compensatory-affective use—will be crucial to understand health-outcomes of GenAI use.

Despite the controversies in this young research field, still the call should go to the industry to create safer products. As the industry aims to repeat the social media story with AI companions, by earning money via a surveillance capitalism mentality and the roll out of ads, it is questionable if we will see safer products promoting the health of users anytime soon. This cries again for the regulators to step up. Finally, we wish for product development to bring the user to the developer’s table. AI companions are built mostly with a “one size fits all” mentality. Considering neurodiversity of people and their varying needs would clearly result in improved digital products [10]. As a result, this would create technology that is less burdensome and be better for people’s health.

## Data Availability

No datasets were generated or analysed during the current study.
